# Supplementary material for: Chromatinization of Escherichia coli with archaeal histones
Source: eLife. 2019 Nov 6;8:e49038. doi: 10.7554/eLife.49038 (PMC6867714; doi:10.7554/eLife.49038)
Supplement: Supplementary file 1. [file elife-49038-supp1.docx]

**Supplementary File 1. E. coli K-12 MG1655-derived strains constructed for this study**

| **Strain name** | **Plasmid name** | **Insert sequence (histone CDS)** |
| --- | --- | --- |
| Ec-hmfA | pD681-hmfA | ATGGGCGAGCTGCCAATTGC  GCCGATCGGCCGCATTATCA  AAAATGCCGGTGCGGAGCGT  GTGAGCGACGACGCACGTAT  CGCGCTGGCAAAGGTTCTGG  AAGAAATGGGTGAAGAAAT  TGCCTCCGAAGCTGTCAAAT  TGGCAAAACACGCGGGTCGT  AAGACGATCAAAGCCGAAGA  TATCGAGCTGGCGCGCAAAA  TGTTTAAGTAA |
| Ec-hmfB | pD681-hmfB | ATGGAACTGCCAATTGCCCC  TATCGGTCGTATTATTAAAG  ACGCTGGTGCCGAGCGCGTG  AGCGATGACGCGCGCATCAC  CCTGGCAAAGATTCTGGAAG  AAATGGGCCGTGACATTGCG  TCCGAGGCCATCAAACTGGC  ACGTCACGCGGGTCGTAAGA  CGATCAAAGCTGAAGATATC  GAGCTGGCAGTTCGTCGCTT  CAAAAAGTGA |
| Ec-EV | pD681 | no insert |
| Ec-hmfA_nb_ | pD681-hmfA_nb_ | ATGGGCGAGCTGCCGATTGC  GCCGATTGGTCGTATTATCA  CCAACGCTGGCGCGGAGAGC  GTTTCCGACGACGCGCGCAT  TGCATTGGCAAAGGTCCTGG  AAGAAATGGGTGAAGAAATC  GCAAGCGAAGCCGTGAAACT  GGCGAAACACGCGGGTCGTA  AGAAAATCAAAGCTGAAGAT  ATCGAGCTGGCCCGTAAAAT  GTTCAAGTAA |
| Ec-hmfB_nb_ | pD681-hmfB_nb_ | ATGGAACTGCCGATTGCGCC  GATCGGCCGCATTATCACCG  ACGCGGGTGCCGAGAGCGTG  AGCGATGACGCACGCATCAC  GCTGGCGAAGATTCTGGAAG  AAATGGGCCGTGACATCGCG  TCCGAGGCCATTAAACTGGC  ACGTCACGCGGGTCGTAAAA  AGATCAAAGCTGAAGATATT  GAGTTGGCAGTTCGCCGTTT  CAAGAAATAA |
