# Supplementary material for: Chromatinization of Escherichia coli with archaeal histones
Source: eLife. 2019 Nov 6;8:e49038. doi: 10.7554/eLife.49038 (PMC6867714; doi:10.7554/eLife.49038)
Supplement: Supplementary file 2. [file elife-49038-supp2.docx]

**Supplementary File 2. Fourier filtering parameters**

**Sample Growth Phase Fragment size (bp) PcVal Thr (Zsc) # of peaks**

Ec-hmfA Exponential 55 – 65 0.02 0.25 27049

Ec-hmfA Exponential 85 - 95 0.015 0.25 18983

Ec-hmfA Exponential 115 – 125 0.0125 0.25 15795

Ec-hmfA Exponential 145 - 155 0.0075 0.25 7050

Ec-hmfB Exponential 55 – 65 0.0225 0.25 28023

Ec-hmfB Exponential 85 - 95 0.0175 0.25 25174

Ec-hmfB Exponential 115 - 125 0.0125 0.25 15590

Ec-hmfB Exponential 145 - 155 0.01 0.25 14363

Ec-EV Exponential 55 - 65 0.015 0.25 21375

Ec-EV Exponential 85 - 95 0.015 0.25 12923

Ec-EV Exponential 115 - 125 0.01 0.25 9015

Ec-EV Exponential 145 - 155 0.0075 0.25 2800

Ec-hmfA Stationary 55 - 65 0.02 0.25 22865

Ec-hmfA Stationary 85 - 95 0.0125 0.25 15287

Ec-hmfA Stationary 115 - 125 0.01 0.25 10936

Ec-hmfA Stationary 145 - 155 0.0075 0.25 7608

Ec-hmfB Stationary 55 - 65 0.0225 0.25 17770

Ec-hmfB Stationary 85 - 95 0.0175 0.25 19272

Ec-hmfB Stationary 115 - 125 0.0175 0.25 18240

Ec-hmfB Stationary 145 - 155 0.0175 0.25 18361

Ec-EV Stationary 55 - 65 0.02 0.25 9811

Ec-EV Stationary 85 - 95 0.0175 0.25 12575

Ec-EV Stationary 115 - 125 0.015 0.25 14465

Ec-EV Stationary 145 - 155 0.015 0.25 13274

*M. fervidus* Exponential 55 - 65 0.02 0.25 6418

*M. fervidus* Exponential 85 - 95 0.015 0.25 5176

*M. fervidus* Exponential 115 - 125 0.01 0.25 3707

*M. fervidus* Exponential 145 - 155 0.01 0.25 3576

*M. fervidus* Stationary 55 - 65 0.0225 0.25 8768

*M. fervidus* Stationary 85 - 95 0.0175 0.25 7848

*M. fervidus* Stationary 115 - 125 0.0125 0.25 5208

*M. fervidus* Stationary 145 - 155 0.01 0.25 3798
