# Supplementary material for: Chromatinization of Escherichia coli with archaeal histones
Source: eLife. 2019 Nov 6;8:e49038. doi: 10.7554/eLife.49038 (PMC6867714; doi:10.7554/eLife.49038)
Supplement: Supplementary file 3. [file elife-49038-supp3.docx]

**Supplementary File 3. Outliers in differential expression analysis**

**DEA* pair Growth phase Replicates** excluded**

Ec-hmfA vs Ec-EV Exponential Ec-EV 1

Ec-hmfB vs Ec-EV Exponential Ec-EV 1

Ec-hmfAnb vs Ec-EV Exponential /

Ec-hmfAnb vs Ec-EV Exponential /

Ec-hmfA vs Ec-hmfB Exponential Ec-EV 1

Ec-hmfA vs Ec-hmfAnb Exponential Ec-hmfA 6

Ec-hmfB vs Ec-hmfBnb Exponential /

Ec-hmfA vs Ec-EV Stationary Ec-hmfA 2

Ec-hmfB vs Ec-EV Stationary Ec-hmfA 2

Ec-hmfAnb vs Ec-EV Stationary /

Ec-hmfBnb vs Ec-EV Stationary /

Ec-hmfA vs Ec-hmfB Stationary Ec-hmfA 2

Ec-hmfA vs Ec-hmfAnb Stationary Ec-hmfA 2

Ec-hmfB vs Ec-hmfBnb Stationary Ec-hmfB 1 & Ec-hmfBnb

* DEA: Differential expression analysis

** see NCBI Gene Expression Omnibus accession number GSE127680 for sample IDs
